# SynBioStrainFinder: A microbial strain database of manually curated CRISPR/Cas genetic manipulation system information for biomanufacturing

**DOI:** 10.1186/s12934-022-01813-5

**Published:** 2022-05-14

**Authors:** Pengli Cai, Mengying Han, Rui Zhang, Shaozhen Ding, Dachuan Zhang, Dongliang Liu, Sheng Liu, Qian-Nan Hu

**Affiliations:** 1 CAS Key Laboratory of Computational Biology, Shanghai Institute of Nutrition and Health, University of Chinese Academy of Sciences, Chinese Academy of Sciences, Shanghai, 200031 China; 2 Chemical.AI Wuhan, Wuhan, 430062 China

**Keywords:** CRISPR/Cas system, Gene-editing method, Microorganism, Database, Strain cultivation, Genome sequence, Strain-related compound

## Abstract

**Background:**

Microbial strain information databases provide valuable data for microbial basic research and applications. However, they rarely contain information on the genetic operating system of microbial strains.

**Results:**

We established a comprehensive microbial strain database, SynBioStrainFinder, by integrating CRISPR/Cas gene-editing system information with cultivation methods, genome sequence data, and compound-related information. It is presented through three modules, Strain2Gms/PredStrain2Gms, Strain2BasicInfo, and Strain2Compd, which combine to form a rapid strain information query system conveniently curated, integrated, and accessible on a single platform. To date, 1426 CRISPR/Cas gene-editing records of 157 microbial strains have been manually extracted from the literature in the Strain2Gms module. For strains without established CRISPR/Cas systems, the PredStrain2Gms module recommends the system of the most closely related strain as a reference to facilitate the construction of a new CRISPR/Cas gene-editing system. The database contains 139,499 records of strain cultivation and genome sequences, and 773,298 records of strain-related compounds. To facilitate simple and intuitive data application, all microbial strains are also labeled with stars based on the order and availability of strain information. SynBioStrainFinder provides a user-friendly interface for querying, browsing, and visualizing detailed information on microbial strains, and it is publicly available at http://design.rxnfinder.org/biosynstrain/.

**Conclusion:**

SynBioStrainFinder is the first microbial strain database with manually curated information on the strain CRISPR/Cas system as well as other microbial strain information. It also provides reference information for the construction of new CRISPR/Cas systems. SynBioStrainFinder will serve as a useful resource to extend microbial strain research and application for biomanufacturing.

## Background

The development of genome sequencing and clustered regularly interspaced short palindromic repeats (CRISPR)/CRISPR-associated (Cas) technology has allowed an increasing number of microbial strains with excellent or unique characteristics to be studied and exploited [1–3]. These strains also provide potential chassis options for biological manufacturing [4]. Meanwhile, several databases with information of microorganisms have been published, besides the National Center for Biotechnology Information (NCBI) and strain culture collections. The Global Catalog of Microorganisms gathers strain catalog information [5]. KOMODO collects the culture medium for all bacterial strains and provides possible formulations for strains without established culture media [6]. Cell2Chem includes 40,370 species and 125,212 compounds with microbial strain information [7]. These databases provide valuable information for microbiological research [8, 9]. However, genetic manipulation systems for microbial strains are not frequently reported in these databases.

Genetic manipulation systems are indispensable for basic research and biomanufacturing applications. CRISPR/Cas has become the most well-known and widely used method for gene editing compared with the Cre/loxP recombination system, zinc-finger nucleases (ZFNs), and transcription activator-like effector nucleases (TALENs) [10–13]. The CRISPR/Cas system originates from the adaptive immune system against invading foreign nucleic acids and is widely found in bacteria and archaea [14]. It forms the basis of powerful gene-editing tools [15]. It consists of an endonuclease and tracRNA/crRNA, which is further simplified as a single sgRNA [15]. Cas9 and Cpf1 (Cas12a) are the most extensively studied endonucleases for gene editing [1]. In addition, via modification and fusion, the derived mutant Cas9 nickase and nuclease-deficient Cas9 (with only DNA binding activity but no cleavage activity), which can be used for gene regulation independently or by fusion with other elements, further improve and expand the application of this system in model and non-model microorganisms, enabling CRISPR-mediated epigenome editing, genome/chromatin imaging, and manipulation of chromatin topology [1, 2, 12, 16]. The development of simple, rapid, powerful, and economical CRISPR/Cas9 technologies has introduced a new era for genome editing, and they have been applied in a wide range of fields, including clinical, pharmaceutical, agricultural, food, and energy fields [17–21]. Computational tools and resources supporting CRISPR-Cas experiments have also been developed, including a variety of sgRNA design tools [22–26]. Addgene (https://www.addgene.org/) [27], an international nonprofit plasmid and data resource, can retrieve the CRISPR/Cas plasmids of some but not all microbial strains, and it does not include additional microbial strain information. Comprehensive and convenient methods for retrieving microbial species-related information are lacking. Therefore, the development of a comprehensive database of strains with information on the CRISPR/Cas genetic manipulation system, as well as other relevant information, is needed.

Here, we established SynBioStrainFinder (http://design.rxnfinder.org/biosynstrain/), a knowledge database containing CRISPR/Cas genetic manipulation system information of microbial strains. The cultivation, genome sequences, and compound-related information of all microbial strains were also integrated to facilitate rapid queries for strain-related information in one place. Information can be retrieved using the following three modules: Strain2BasicInfo for cultivation and genome sequence information integrated from several databases, Strain2Gms for CRISPR/Cas genetic manipulation system information manually curated from the literature, and Strain2Compd for strain-related compounds calculated using the term frequency-inverse document frequency (TF-IDF) method. It also provides CRISPR/Cas system information for the most closely related strains as a reference to facilitate new construction in the PredStrain2Gms module. SynBioStrainFinder provides a useful and comprehensive one-stop microbial strain data resource with a user-friendly interface for querying, browsing, and visualizing detailed information about the CRISPR/Cas system, as well as other microbial strain properties. To facilitate the application of strain information in a simple and intuitive way, all microbial strains are labeled with stars based on the order and availability of data for culture media, genome sequencing, genetic manipulation systems, and strain-related compounds. We expect this database to serve as an important resource and extend the utilization of microbial strains by microbiologists and synthetic biologists.

## Results

### Database summary

**Fig. 1:**
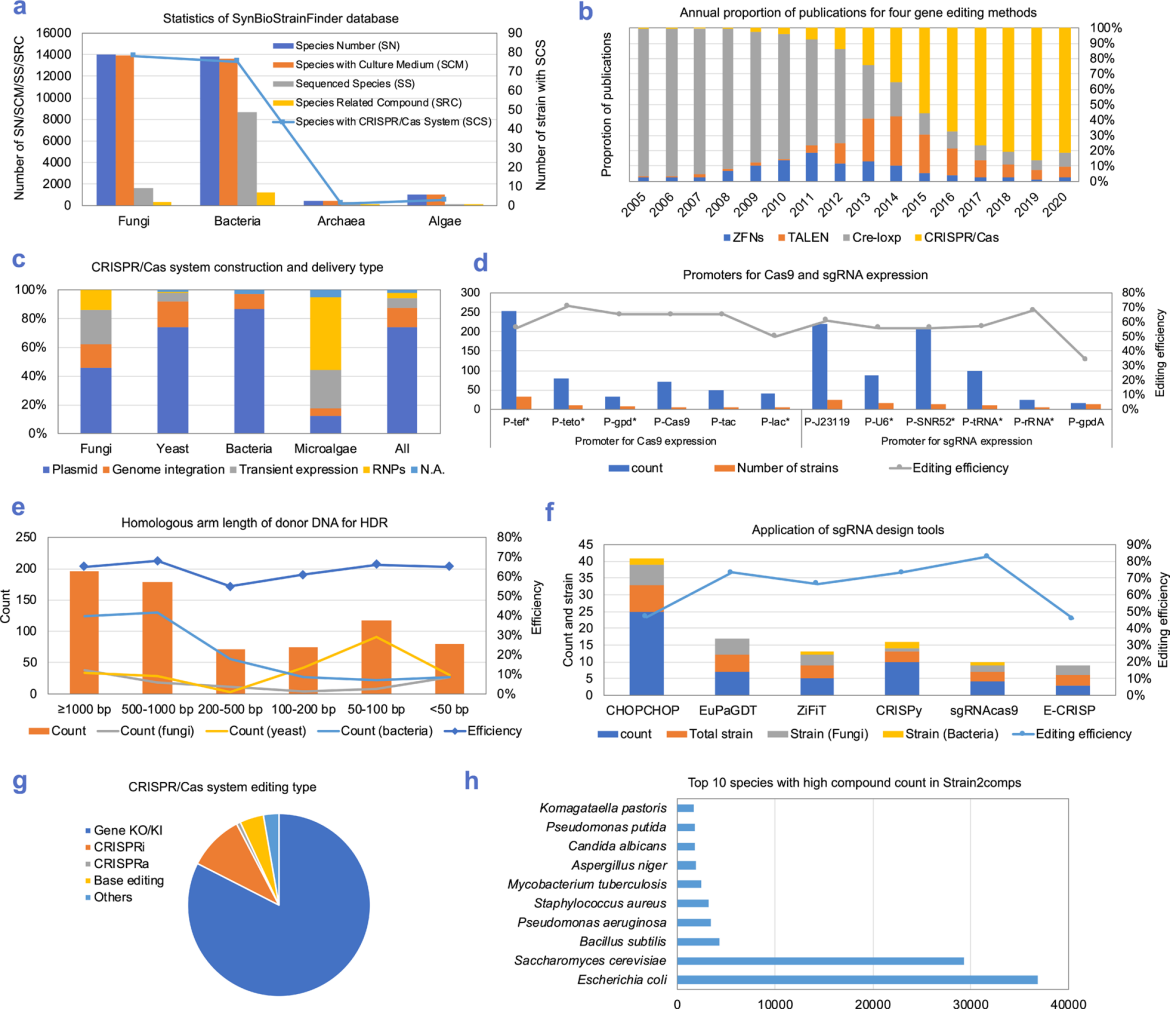
Statistical summary of the SynBioStrainFinder database. **a** Main data in SynBioStrainFinder, including species number (SN), species with culture medium (SCM), sequenced species (SS), species with reported compounds (SC), and species with CRISPR/Cas systems (SCS). **b** Proportions of publications reporting four gene-editing methods (zinc-finger nucleases (ZFNs), transcription activator-like effector nucleases (TALENs), Cre/loxp, and CRISPR/Cas) annually. **c** CRISPR/Cas system construction and delivery type. **d** Frequently used promoters for Cas9 and sgRNA expression. *, corresponding promoter and its deformation. **e** Homologous arm length of donor DNA for HDR. **f** Commonly used sgRNA design tools. **g** CRISPR/Cas system gene-editing type. **h** Top 10 species with high strain-related compound counts

SynBioStrainFinder is the first database of microbial strain information with manually curated data for the CRISPR/Cas gene-editing method, as well as strain cultivation, genome sequencing, and strain-related compounds. It consists of three modules, namely, Strain2BasicInfo for genome sequence data and basic information, Strain2Gms/PredStrain2Gms containing information on the CRISPR/Cas genetic manipulation system for each strain or providing a reference for strains without an established CRISPR/Cas gene-editing system, and Strain2Compd providing strain-related compounds. To date, SynBioStrainFinder contains information for 32,320 species, including 16,404 fungi, 14,072 bacteria, 483 archaea, and 1361 algae. There are 139,499 records of strain growth, 1426 records of CRISPR/Cas systems, and 773,298 records of 1768 microbial strains with compound information in Strain2BasicInfo, Strain2Gms, and Strain2Compd, respectively. In SynBioStrainFinder, 11.4% of fungi, 62.8% of bacteria, 69.0% of archaea, and 9.6% of algae have sequenced genomes. Up to June 2020, 157 microbial species had an established CRISPR/Cas gene-editing system. In addition, 4.9% of fungi (78), 0.9% of bacteria (75), 0.3% of archaea (1), and 3.0% of algae (3) among taxa with sequenced genomes had a CRISPR/Cas genome editing system (Fig. 1a).

The CRISPR/Cas gene-editing method is the most popular genetic manipulation method (Fig. 1b). In the Strain2Gms module, we evaluated the delivery type, editing type, promoters used for Cas9 and sgRNA expression, homologous arm length of donor DNA for Homology directed repair (HDR), and commonly used sgRNA design tools. (1) Based on the current database statistics, plasmid (74%) is the main construction and delivery mode, followed by genomic integration (13%), transient expression (7%), and ribonucleoproteins (RNPs) (4%) (Fig. 1c). (2) The most frequently used promoters for Cas9 expression are the tef [28–32] and teto promoters [33–36] in fungi and bacteria, respectively. For sgRNA expression, the commonly used promoters are the SNR52 [30, 37] and U6 promoters [28, 38] in fungi and the J23119 promoter [39, 40] in bacteria (Fig. 1d). (3) For precise editing by HDR, the average lengths of repair templates for fungi, yeast, and bacteria are 567, 276, and 612 bp, respectively. Templates in the length range of 200–500 bp are the least used and have relatively low editing efficiency. Templates of 50–100 in length bp are the most commonly used for yeast. Bacteria usually use longer homologous arms (Fig. 1e). The length selection of the homologous arm of the donor DNA usually depends on the intrinsic DSB repair mechanism of the strain. (4) CHOPCHOP [41–43] is the most commonly used sgRNA design tool (Fig. 1f). To facilitate sgRNA design, we selected and updated the webserver tools from WeReview [24] as CRISPR tools on the home page of SynBioStrainFinder. (5) In the current database, the main editing types are CRISPR-based gene knockout/knockin (83%) and CRISPR interference (CRISPRi) (10%) (Fig. 1g). In addition to the factors mentioned above, other information related to the CRISPR/Cas system, including edited genes, selection markers, and transformation methods, can be retrieved from the database if this information is included in the literature.

There are 773,298 records of compound information corresponding to 1768 microbial strains in the Strain2Compd module. Out of the top 10 species with the highest counts of the corresponding compounds, six strains are traditional industrial microorganisms used in biomanufacturing, namely *Escherichia coli* [44], *Saccharomyces cerevisiae* [45], *Bacillus subtilis* [46, 47], *Aspergillus niger* [48], *Pseudomonas putida* [49–51], and *Komagataella pastoris* [52, 53]. *Escherichia coli* and *S. cerevisiae* are the most extensively studied strains, which is also reflected by a higher number of related compounds for these strains than for other strains. The other four species are pathogens, namely *Pseudomonas aeruginosa* [54], *Staphylococcus aureus* [55], *Mycobacterium tuberculosis* [56], and *Candida albicans* [57] (Fig. 1h).

**Fig. 2:**
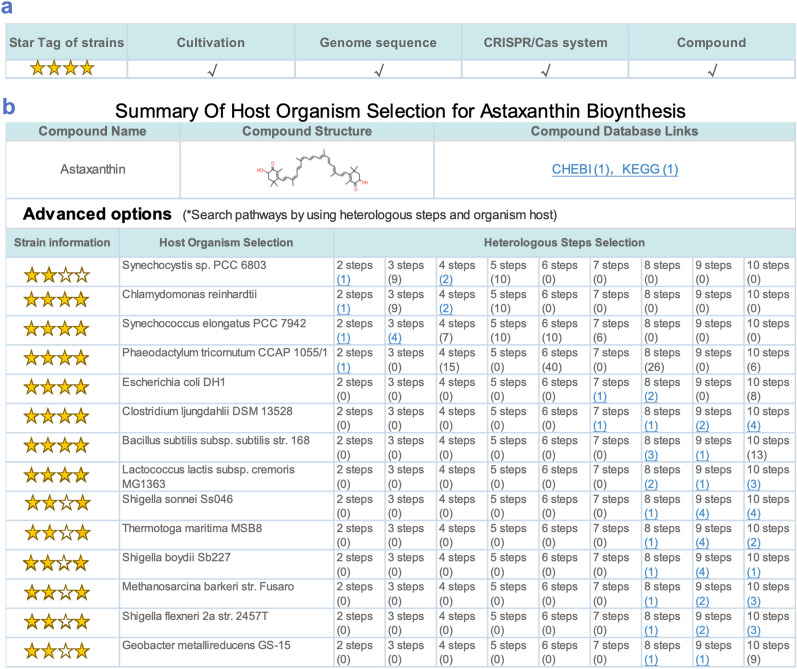
Application of SynBioStrainFinder data. **a** Example of star-tags for strains in SynBioStrainFinder. All microbial strains were marked with stars according to the order and extent of information available on strain cultivation, genome sequence, CRISPR/Cas genetic operating system, and related compounds. **b** Application of star-tags to host organism selection with CF-targeter. The strain star-tag can be added next to the strain list

SynBioStrainFinder provides a user-friendly interface for querying, browsing, and visualizing detailed information on microbial strains, especially the CRISPR/Cas gene-editing system. Furthermore, to obtain information of these strains in a simple and intuitive manner, all microbial strains were marked with stars according to the order and extent of information available on strain cultivation, genome sequence, CRISPR/Cas genetic operating system, and related compounds. Star-level information can be used for microbial strain selection. For example, it can be applied to the CF-targeter [58], which is a web server for host organism selection of biosynthetic pathway design. In the search interface for host organism selection for astaxanthin biosynthesis, by adding a strain star-tag next to the strain list, we can clearly and intuitively obtain information about the corresponding strain, thereby assisting strain selection (Fig. 2).

### User interface

**Fig. 3:**
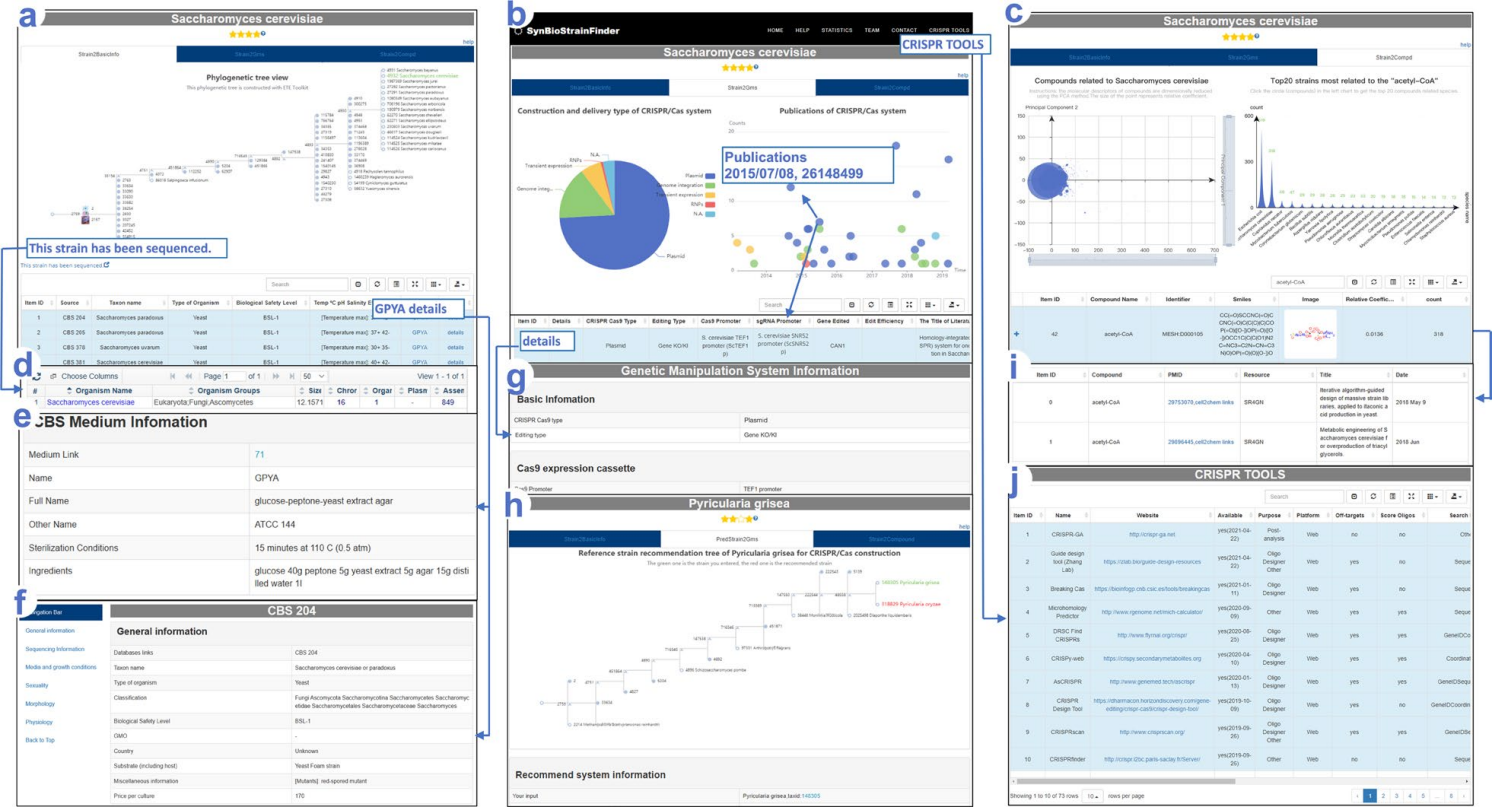
Database content and interface of SynBioStrainFinder. The database consists of three modules: **a** Strain2BasicInfo module for strain cultivation and genome sequence, **b** Strain2Gms/PredStrain2Gms module for CRISPR/Cas genetic operating system, and **c** Strain2Compd module for related compounds. The top of the browse-results page shows statistical information for the Strain2Gms and Strain2Compd modules and the phylogenetic tree for the Strain2BasicInfo module. **d**–**j** The bottom of the browse-results page contains detailed information, which can be expanded further

Users can quickly browse the database using the Latin name of the species on the first page. First, stars indicating the availability of information about the strain are shown at the top of the retrieved page immediately below the strain name. The following are the three modules: Strain2BasicInfo, Strain2Gms/PredStrain2Gms, and Strain2Compd (Fig. 3a–c). The top of the browse-results page shows statistical information for the Strain2Gms and Strain2Compd modules and the phylogenetic tree for the Strain2BasicInfo module. The bottom of the browse-results page contains detailed information (Fig. 3a–c). SynBioStrainFinder also supports retrieval using the strain’s generic name. All strains in this genus will be displayed below the search box on the first browse page, from which specific strains can be selected.

On the Strain2BasicInfo browse page, the strain genome sequence (Fig. 3d), as well as culture medium and conditions, can be obtained by clicking “detail” to enter the detailed information page (Fig. 3e, f). On the Strain2Gms browse page, the CRISPR/Cas construction type, publication time, and numbers are counted and shown at the top of the page. Clicking on each statistical color block or dot displays the details in the detailed data entry at the bottom of the page (Fig. 3b). The detailed CRISPR/Cas information includes the Cas9 and sgRNA expression plasmids, promoters, and terminators (Fig. 3g). In addition, the most commonly used CRISPR/Cas tools are shown in the navigation bar on the first page (Fig. 3j). For strains without an established CRISPR/Cas system, the PredStrain2Gms module appears instead of the Strain2Gms module to show the reference information (Fig. 3h). On the Strain2Compd browse page, the upper left shows the statistics for retrieving compounds associated with the strain. The upper right shows the statistical information for strains associated with the selected compound. An example of compound information is shown in Fig. 3c, i.

## Discussion

Genetic operating systems play an important role in the research and application of microorganisms. With the rapid development and application of CRISPR/Cas systems, a growing number of distinctive microbial strains could be developed into microbial cell factories. Nevertheless, most of the current microbial strain databases do not include information about genetic operating systems. Therefore, we built the SynBioStrainFinder database, which includes information on the most common CRISPR/Cas gene-editing system corresponding to all microbial strains. Furthermore, it provides a reference strain with an established CRISPR/Cas system for the construction of new systems. We also integrated cultivation, genome sequence, and compound information simultaneously to facilitate the rapid retrieval of microbial strain information in one place.

Although only 157 microbial strains have reported CRISPR/Cas gene-editing systems through the statistics of SynBioStrainFinder, the range of strains with genetic operating systems has expanded substantially. Among the 157 strains with established CRISPR/Cas gene-editing systems, only 29% utilize the Cre/loxP method, illustrating this point. Shared features of the CRISPR/Cas gene-editing method are among different species in the same genus, to a certain extent, although the method of some species requires slight modifications. Accordingly, we developed the PredStrain2Gms module based on the evolutionary relationships among strains with and without genome editing systems, providing a basis for the construction of a new system. Owing to the relatively small number of strains building CRISPR/Cas systems, PredStrain2Gms may only provide a useful reference for a limited number of strains. The statistical analysis of data in the Strain2Gms module might also provide some suggestions for new system construction, which includes the delivery method, Cas9 and sgRNA expression promoter, homologous arm of HDR, and sgRNA design tool. Although we provide the editing efficiency, it is affected by many factors, including the characteristics of the target genes in addition to the CRISPR/Cas system itself. Additionally, the size of the dataset affects the statistical analysis. Therefore, when building a new system, it is necessary to make reasonable attempts based on the characteristics of microbial strains.

Statistical results show that plasmids are the most commonly used method among the four CRISPR/Cas delivery methods, each of which has distinct advantages and disadvantages [2, 16]. For plasmid delivery, Cas9 and sgRNA are constructed in one or two plasmids, which are usually designed to enable curing for subsequent gene editing, such as by using the temperature-sensitive replicons repA101, pSG5, repF, and pBL1^ts^ [40, 59–64]. Plasmid delivery, transient expression, and genomic integration require the effective expression of Cas9 and/or sgRNA, although sgRNA can also be transcribed or synthesized in vitro. The search and optimization of suitable expression regulatory elements can be time-consuming and expensive. The RNPs method is more suited for the CRISPR/Cas system development of relatively new hosts with limited genetic manipulation tools or without a foundation. Owing to the structural complexity of fungi, fairly diverse construction and delivery methods are used relative to bacteria during CRISPR/Cas system construction, and the availability of plasmids, selection of the nuclear localization sequence, and identification of type III promoter and/or promoter effectiveness should be considered [2, 3]. Therefore, in the Strain2Gms module, we ensured that at least one detailed CRISPR/Cas record was provided for each species and relatively more information for fungal CRISPR/Cas systems was provided. Bacteria are considerably simpler than fungi because they mostly contain plasmids and one type of RNA polymerase promoter [1]. For the selection of sgRNA design tools, those offering more than one scoring algorithm to accurately assess gRNA activity are preferable, such as CRISPOR [65]. The usage range should also be considered, as some sgRNA software models are not universally applicable. For example, the Moreno-Mateos score is best suited for experiments with gRNAs expressed in vitro [23]. In this version of the database, we collected basic gene-editing types, including gene knockouts, knock-ins, base editing, CRISPRi, and CRISPRa, which are the most used initially.

Owing to the limitations of the CRISPR/Cas data collection method and the limited strains with constructed CRISPR/Cas gene-editing systems, the volume of data in Strain2Gms is relatively small at present. It will be updated continuously for improvement. Furthermore, to further facilitate information acquisition and, thus, promote the research and utilization of microbial strains, other information on microbial strains will be added, such as the plasmids, promoters, metabolic network models, various omics data, and product information. Nevertheless, SynBioStrainFinder is a useful database to facilitate new CRISPR/Cas system construction, providing abundant and concentrated microbial strain information for chassis construction and basic research, as well as a variety of chassis recommendations for biomanufacturing. In addition, labeling strains with stars allows a simple, intuitive, and convenient-for-use display of strain information in strain selection tools.

## Conclusions

SynBioStrainFinder is the first database with manually curated information on the microbial strain CRISPR/Cas system, the most widely used genetic manipulation method. It provides a reference strain with an established CRISPR/Cas system for the construction of new CRISPR/Cas systems. The database also comprises other microbial strain information (cultivation methods, genome sequence data, and strain-related compound information) to facilitate rapid strain information queries. Tagging stars to indicate strain information provides a simple, intuitive basis for microbial strain selection. SynBioStrainFinder will continue to expand, aiming to serve as an important resource to extend microbial strain research and application for biomanufacturing by microbiologists and synthetic biologists.

## Methods

### Data collection and database content

The CRISPR/Cas system information in the Strain2Gms module was manually curated from the literature. Other information was processed and compiled from public resources, including NCBI [66], DSMZ (https://www.dsmz.de/), CBS (https://wi.knaw.nl/), UTEX collection (https://utex.org), Global Catalog of Microorganisms [5, 67], and Cell2Chem [7]. The basic strain information in Strain2BasicInfo includes the strain name, taxon, safety level, and culture medium and conditions from the CBS, DSMZ, and UTEX databases, as well as genome sequence information from NCBI [66]. Links to external resources were also provided, including PubMed ID, genome sequencing in NCBI, sgRNA design tools, and chemical ID in PubMed.

### Strain2Gms/PredStrain2Gms module construction

For CRISPR/Cas genetic manipulation information, all publications up to June 2020 matching the keyword “CRISPR*” and generic names for taxa in the microbial strain list of SynBioStrainFinder were first retrieved from PubMed [66]. A total of 1326 titles and/or abstracts of publications were reviewed to obtain microbial-related CRISPR/Cas systems. After further filtering, 472 publications related to the construction of a CRISPR/Cas tool for microbial strains were retained for a detailed review of the full text to extract CRISPR/Cas construction-related information. The manually extracted information included the species name, PubMed ID for the publication, CRISPR/Cas editing types (Gene KO/KI, CRISPRi, CRISPRa, base editing, and others), CRISPR/Cas construction and delivery types (plasmid, RNPs, transient expression, and genome integration), CRISPR/Cas editing targets (DNA, RNA, chromosome, and others), CRISPR/Cas system details (Cas9 and sgRNA expression plasmid or expression cassette, and related promoter, terminator, selection marker, and donor DNA for homologous recombination repair (HDR)), CRISPR/Cas editing targets and editing efficiency, and sgRNA design tools.

If a CRISPR/Cas gene-editing system is not available for a retrieved strain, the PredStrain2Gms module replaces Strain2Gms. In this module, the CRISPR/Cas gene-editing system of the most closely related strain is recommended as a reference. Using the ETE Toolkit (3.0) [68], a phylogenetic tree for all strains according to the strain taxonomy ID was constructed to identify the most closely related strain. If similar relationships to multiple strains are detected, the most suitable strain will be selected by an exhaustive coefficient, which is an index reflecting the completeness of items of the CRISPR/Cas system for strains in our library, such as Cas9 marker, sgRNA marker, and editing efficiency information.

### Strain2Compd module construction

In the Strain2Compd module, a weighted statistical method, TF-IDF, was used to find the most relevant compound for the target strain. We first extracted all abstracts of articles obtained in which searches of the strain and its relevant compound co-occurred to form a total abstract text set composed of multiple independent article abstracts (N). The TF value of each compound was then calculated, which refers to the frequency at which each compound appears in the total abstract text. Second, the IDF value for each compound was calculated, which was the total number of article abstracts (N) divided by the number of articles containing the compound. Finally, the product of TF and IDF yielded the relative coefficient for each compound. We calculated the correlation coefficient for all compounds related to the strain of interest using the TF-IDF algorithm. Larger correlation coefficients for compounds indicated higher relevance of the strain of interest.

$$For \; a \; term \; i \; in \; document \; j{:}$$

$${W }_{i,j}={tf }_{i,j} \times \mathrm{log}\left(\frac{N}{{df}_{i}}\right)$$

$${tf }_{i,j}=number \; of \; occurrences \; of \; i \; in \;j$$

$${df}_{i}=number \; of \; documents \; containing \; i$$

$$N=total \; number \; of \; document$$

### System design and implementation

The entire project was conducted using Ubuntu (version 18.04.2). Python (version 3.6.8) and Django (version 1.11.7) were used to build SynBioStrainFinder and the interactive interface. The data for the entire project was stored in MySQL (version 8.0.16). ECharts (version 4.2.0; http://echarts.baidu.com) was used as a graphical visualization framework. Bootstrap Table (version 1.15.5) was used for the static and dynamic display of data tables, which relies on Bootstrap (version 3.3.7) and jQuery (version 2.1.1). A modern web browser that supports HTML5, such as Google Chrome, Firefox, Safari, Opera, or IE 9.0+, is recommended. SynBioStrainFinder is freely available to the research community using the web link provided (http://design.rxnfinder.org/biosynstrain/). Users are not required to register or login to access the features in the databases.

## Data Availability

Not applicable.

## Abbreviations

Cas: CRISPR-associated
CRISPR: Clustered regularly interspaced short palindromic repeats
CRISPRi: CRISPR interference
HDR: Homology-directed repair
NCBI: National Center for Biotechnology Information
RNPs: Ribonucleoproteins
TALEN: Transcription activator-like effector nuclease
TF-IDF: Term frequency-inverse document frequency
ZFN: Zinc-finger nuclease
